# Extending the Aerolysin Family: From Bacteria to Vertebrates

**DOI:** 10.1371/journal.pone.0020349

**Published:** 2011-06-08

**Authors:** Pawel Szczesny, Ioan Iacovache, Anna Muszewska, Krzysztof Ginalski, F. Gisou van der Goot, Marcin Grynberg

**Affiliations:** 1 Institute of Biochemistry and Biophysics, Polish Academy of Sciences, Warsaw, Poland; 2 Institute of Experimental Plant Biology, University of Warsaw, Warsaw, Poland; 3 Faculty of Life Sciences, Global Health Institute, Ecole Polytechnique Fédérale de Lausanne, Lausanne, Switzerland; 4 Interdisciplinary Centre for Mathematical and Computational Modelling, University of Warsaw, Warsaw, Poland; Baylor College of Medicine, United States of America

## Abstract

A number of bacterial virulence factors have been observed to adopt structures similar to that of aerolysin, the principal toxin of *Aeromonas* species. However, a comprehensive description of architecture and structure of the aerolysin-like superfamily has not been determined. In this study, we define a more compact aerolysin-like domain – or aerolysin fold – and show that this domain is far more widely spread than anticipated since it can be found throughout kingdoms. The aerolysin-fold could be found in very diverse domain and functional contexts, although a toxic function could often be assigned. Due to this diversity, the borders of the superfamily could not be set on a sequence level. As a border-defining member, we therefore chose pXO2-60 – a protein from the pathogenic pXO2 plasmid of *Bacillus anthracis*. This fascinating protein, which harbors a unique ubiquitin-like fold domain at the C-terminus of the aerolysin-domain, nicely illustrates the diversity of the superfamily. Its putative role in the virulence of *B. anthracis* and its three dimensional model are discussed.

## Introduction

Among bacterial toxins, the most widely distributed and largest family is of pore-forming toxins (PFTs). Within the PFT family, the largest sub-family is currently composed of the Cholesterol Dependent Cytolysins (CDCs), which are produced by Gram-positive bacteria of *Bacillus*, *Clostridium*, *Streptococcus*, *Listeria* and *Arcanobacterium* genera. Interestingly, structural, but not sequence, similarity have recently been found with proteins produced by the vertebrate immune system, more specifically with the C8 and C9 components of the complement cascade and with perforin [1]–[3]. These latter proteins all contain a so called MACPF domain, and have a structure very similar to that of CDCs. Perforin like proteins are also found in Protozoans and in particular in the human parasites *Toxoplasma gondii* and *Plasmodium falciparum* [1].

A second family has started to emerge as new structures of PFTs are being discovered: the aerolysin family. Aerolysin is produced by *Aeromonas* species, but related proteins are present in both Gram-positive and Gram-negative bacteria, plants and eukaryotes. It shares high sequence identity with alpha-toxin from *Clostridium septicum* [4], as well as with enterolobin produced by the seeds of the Brazilian tree *Enterolobium contorliquum* [5], [6]. The aerolysin family was subsequently extended, based on the analysis of conserved motifs [7], to hydralysins produced by *Cnidaria*, ε-toxin form *Clostridium perfringens* [8], a hemolytic lectin from the parasitic mushroom *Laetiporus sulphureus* [9] and structurally to parasporin-2 from *Bacillus thuringiensis* [10]. These similarities are reflected in protein structure classifications, such as SCOP or CATH, in which aerolysins and epsilon toxins (in case of SCOP) belong to the same superfamily.

These various toxins are thought to share the same overall mode of action. The toxin is produced by the bacterium as a soluble protein that can either be a precursor, as is the case for aerolysin itself. The aerolysin precursor is called proaerolysin and activation consists in the proteolytic processing of a C-terminal peptide [11]. The soluble toxin diffuses towards its target cell where it binds via specific surface receptors, which are GPI-anchored proteins for aerolysin and *C. septicum* alpha-toxin, but likely to differ for other members of the family. Once receptor bound and proteolytically activated, the toxin undergoes circular polymerization, generating ring like structures that subsequently insert into the membrane and form a pore. While aerolysin and e-toxin form heptamers, the stochiometry might differ between members. The membrane-inserting portion represents only a small fraction of the entire protein. It is thought to cross the membrane in ß-barrel conformation, as shown for the leukocidin-like fold alpha hemolysin of *Staphylococcus aureus*. The sequence of the transmembrane domain of aerolysin family pore-forming toxins is thus characterized by the alternating pattern of polar and hydrophobic residues, rather than any distinct sequence conservation [11].

Here we analyzed the similarities between various aerolysin like structures and defined the „aerolysin domain.” Searching through multiple genomes for proteins containing this domain, we reveal that members of the superfamily can be found in all kingdoms. Sequence variability and fusion events with other domains suggest aerolysin core is widely used and may serve for diverse functions.

## Results

### Determination of the conserved core of the aerolysin domain

**Figure 1:**
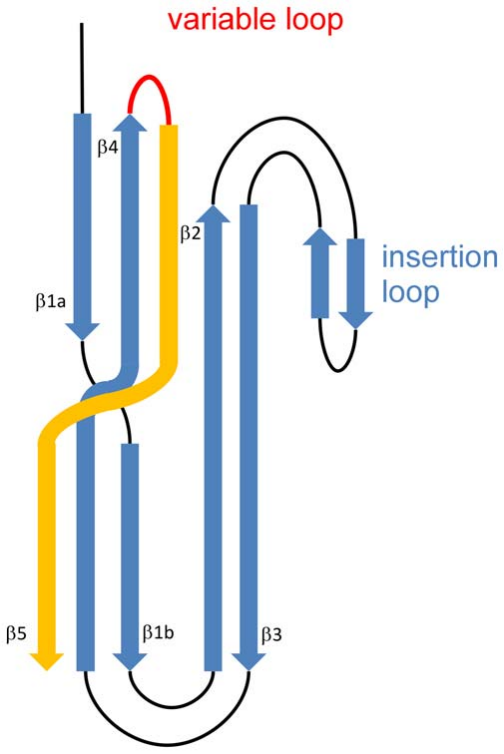
A simplified topology of a common core for aerolysin-like β-PFTs. Five β-strands that span the structure of common core of aerolysin-like toxins have been numbered from 1 to 5. The first β-strand in most cases does not maintain extended secondary structure through, therefore we divided it into two (denoted as β1a and β1b). β-strands in so-called “insertion” loop are not strictly preserved, so we did not number them. The fifth strand (marked in orange) is not present in the alignment. Due to different lenghts of the variable loop connecting β4 and β5, we were unable to precisely align the last strand (see text).

**Figure 2:**
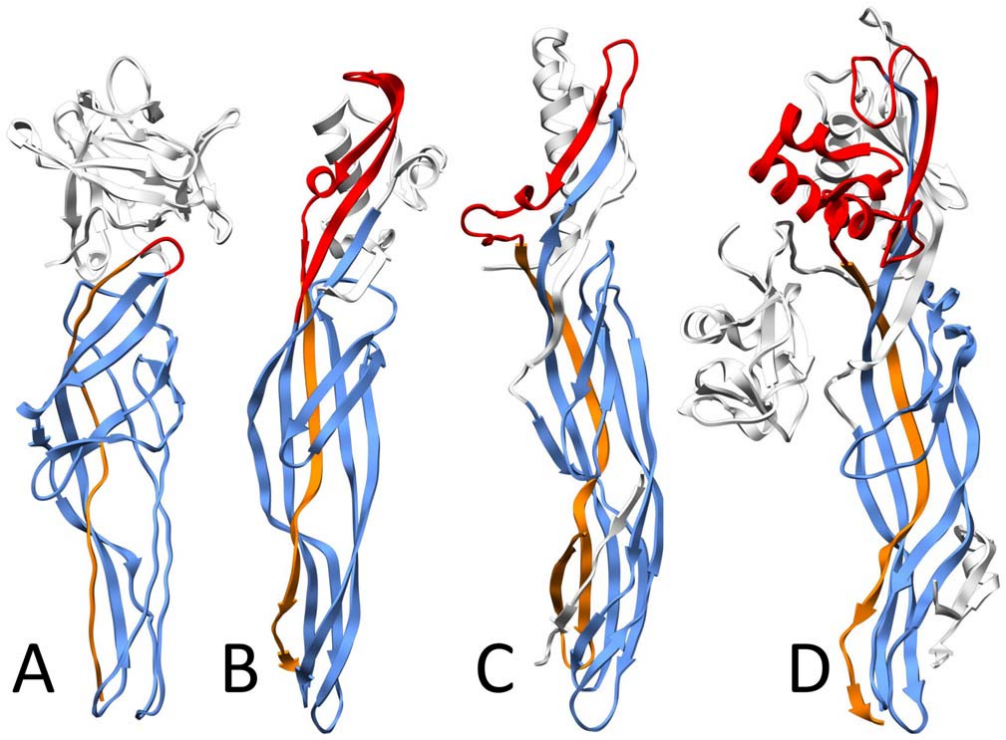
Topology of a common core for aerolysin-like β-PFTs mapped onto structures. **A**: hemolytic lectin, PDB code 1w3f; **B**: parasporin, PDB code 1ztb; **C:** epsilon-toxin, PDB code 1uyj; **D:** proaerolysin, PDB code 3c0n. Color scheme as on Fig. 1: blue - core with conserved sequence; red – variable loop; orange – the fifth, weakly conserved β-strand (see text).

**Figure 3:**
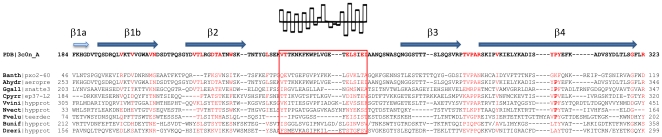
Alignment of the conserved common core of aerolysin like β-PFTs. Beta strands corresponding to the structure of proaerolysin (3c0n) and previously shown topology are marked above the alignment. The B1a is marked in light blue as only part of it seem to hit the alignment. A putative “insertion loop” is marked with red box. This fragment has calculated an average hydrophobicity according to Kyte-Doolittle scale in each column and shown above the alignment. Abbrevations: **Banth**|pXO2-60 - pXO2-60 protein, gi: 51704196 [*Bacillus anthracis*]; **PDB**|3c0n_A - proaerolysin, PDB structure 3c0n, chain A [*Aeromonas hydrophila*]; **Ahydr**|aeropre - aerolysin-3 precursor, gi: 2501301 [*Aeromonas hydrophila*]; **Ggall**|snatte3 - protein similar to nattering-3, gi: 118105776 [*Gallus gallus*]; **Cpyrr**|ep37-L2 - ep37-L2 protein, gi: 2339973, [*Cynops pyrrhogaster*]; **Vvini**|hypprot - hypothetical protein, gi: 147838248 [*Vitis vinifera*]; **Nvect**|hypprot - hypothetical protein, gi: 156349328 [*Nematostella vectenensis*]; **Fvelu**|teerdec - TEER-decreasing protein, gi: 3551186 [*Flammulina velutipes*]; **Bunif**|hypprot - hypothetical protein, gi: 160892167 [*Bacteroidetes uniformis*]; **Dreri**|hypprot - hypothetical protein, gi: 162139040 [*Danio rerio*].

Following the procedure described in the method section, we identified 338 sequences in the NCBI non-redundant database that have a detectable similarity to the aerolysin domain. When performing the alignment of these sequences, a subset of which is shown in Fig. 1, we could identify the following conserved common core: two β-strands β1 and β2 followed by what in aerolysin corresponds to the membrane insertion β-hairpin, again followed by two additional β-strands β3 and β4. As observed in the structures (Fig. 2), the first β-strand usually does not adopt a β conformation along its whole length, which was denoted as separation into two individual β -strands numbered as β1a and β1b. Interestingly, the best preserved pattern in the alignment was that of the insertion loop, where polar and non polar residues alternated in all depicted structures (Fig. 3) suggesting that this loop might, as for aerolysin, cross the membrane in a β-hairpin conformation upon oligomerization into a β-barrel structure.

In the aerolysin structure, these four β-strands and the insertion β-hairpin are part of the so-called domains 3 and 4, which respectively form a twisted anti-parallel beta-sheet with an amphipatic β-hairpin and a beta-sandwich (Fig. 2D). The polypeptide chain traverses the boundary between these 2 structural domains five times. Based on the analysis of the four known structures (Fig. 2), one would predict that the aerolysin domain consists of five β-strands, with an insertion loop between strands 2 and 3 and a variable loop between strands β4 and β5 which can range from a few residues, as in the hemolytic pore-forming lectin from *L. sulphureus*, to multiple secondary structure elements such as in aerolysin (Fig. 3).

Our sequence analysis however reveals that what we define as the “aerolysin domain” is shorter, by 100 residues, than the publicly available domain definition, explaining why establishing similarity between all aerolysin-like domains was not an easy task. Indeed even when we removed out the variable loop between β4 and β5, we were unable to construct a reliable alignment consisting of all five major β-strands. Reliable similarity (depicted by blue strands) ended at the β4; further similarities along the sequence (between sequences of different β5s) were only due to biased, beta-strand-like, residue composition of that segment. This is probably due to different evolutionary pressure on that element: presence of a variable loop suggests that this strand is susceptible to phase shifts in the sequence, i.e. to shifts of amino acids along the β-strand as it needs to adapt to the presence of different insertions in the loop (although such shifts are unlikely to alter protein function). Additionally, conservation of this element might not be needed due to stable structural scaffold provided by hairpins β1∶β4 and β2∶β3. Thus, the middle strand of the fold is the necessary part of the topological unit, but it does not belong to highly conserved sequence core. We use the historical convention (domains 3 and 4) and our convention interchangeably. When talking about aerolysin-like structures as a whole, we use historical convention as it refers to two distinct structural units. However, when we refer to sequence-structure relationship in aerolysin superfamily, we prefer the β1–β5 convention, as it better corresponds to relations between various structural elements.

### Species distribution of the aerolysin domain

Proteins containing the aerolysin domain defined in here were found in all kingdoms of life (Table 1). Approximately 90% of the identified proteins were found in Proteobacteria, Firmicutes and Fungi. Examples of most interesting, not previously known family members are shown in Table 2, while the complete list is attached as Table S1.

**Table 1:** Species distribution of proteins belonging to aerolysin-like superfamily with a number of proteins that have assigned a function.

Phylum	No. of proteins	No. of proteins with assigned function
**Bacteria:**	**225**	**193**
- Actinobacteria	7	0
- Bacteroidetes/Chlorobi	3	0
- Cyanobacteria	1	0
- Firmicutes	61	53
- Proteobacteria	151	140
- Tenericutes	2	0
**Archea:**	**3**	**0**
- Euryarcheota	3	0
**Eukaryota:**	**109**	**49**
- Amoebozoa	1	0
- Fungi/Metazoa	100	48
- Viridiplantae	8	1
**Viruses:**	**1**	**1**
- Caudovirales	1	1
**TOTAL:**	**338**	**243**

The functional annotation could be inferred from sequence similarity. Text annotation is not based on experimental evidence (“predicted toxin” or “similar to aerolysin” count as functional annotation; “putative transmembrane protein”, “hypothetical protein” or “predicted protein” are treated as no functional assignment).

**Table 2:** Representatives of previously unknown members of the aerolysin-like superfamily.

Annotation	GI number(s)	Species
hypothetical protein Bsph_1071	169826655	*Lysinibacillus sphaericus*
conserved hypothetical protein	169338027	*Clostridium botulinum*
hypothetical protein SvirD4_00927	256799387	*Streptomyces viridochromogenes*
hypothetical protein pxo2_60	10956450	*Bacillus anthracis*
hypothetical protein HborDRAFT_3048	227882342	*Halogeometricum borinquense*
hypothetical protein HCH_03563	83646295	*Hahella chejuensis*
hypothetical protein MmarC7_0032	150401960	*Methanococcus maripaludis*
predicted protein	154291554	*Botryotinia fuckeliana*
conserved hypothetical protein	255557038	*Ricinus communis*
predicted protein	242210318	*Postia placenta*
unnamed protein product	171678343	*Podospora anserina*
Predicted proteins	169848522, 169850135, 169862541,	*Coprinopsis cinerea*
hypothetical protein	256084754	*Schistosoma mansoni*
hypothetical proteins IscW	241709479, 241644210, 241838790, 241607979, 242002442	*Ixodes scapularis*
yolk proteins 1 and 2	20269940, 20269942	*Hyphantria cunea*
anarchy 1	67848428	*Apis mellifera*
Hypothetical proteins	165972443, 160333564, 156739307, 61651762, 189520990, 160333607, 125820446, 125820386, 189520988, 125820390, 189520992, 125820369, 160333636, 162139040, 125820384, 194579003	*Danio rerio*
Natterin	75571591, 75571593, 75571592, 75571590	*Thalassophryne nattereri*
Natterin-like protein	225714938	*Esox lucius*
Natterin-like protein	213515472	*Salmo salar*
hypothetical proteins	148223884, 168693479, 195972837	*Xenopus laevis*
hypothetical protein BRAFLDRAFT_68290	219416017	*Branchiostoma floridae*
Ep37, gep proteins	2339971, 1169472, 2339973, 6069419, 2339969	*Cynops pyrrhogaster*
PREDICTED: similar to ep37-L2	149419178	*Ornithorhynchus anatinus*
PREDICTED: similar to natterin 3	118105776	*Gallus gallus*

Names represent original NCBI annotations.

Less than 30% of the proteins were annotated as hypothetical of putative proteins. The other 70% had a function confirmed by an experiment or assigned by similarity, and these were almost exclusively from the three groups mentioned before: Proteobacteria, Firmicutes and Fungi A notable exception is a cytotoxin from *Pseudomonas* phage phiCTX, which has an experimentally confirmed toxic function [12]. The presence of a signal sequence was predicted in 139 proteins, around ∼40% of the total, not surprisingly, since many sequences correspond to aerolysin fragments. Also almost 30 (∼30%) sequences, with no assigned function, had a predicted signal peptide.

About half of the eukaryotic and one third of the prokaryotic species harboring aerolysin domain containing proteins are considered non-pathogenic. These bacterial species are aquatic and some pathogenic ones are of the same provenience, e.g. *Aeromonas salmonicida* and *Vibrio splendidus*. In Eukaryotes, except for fungi, cnidarians and the Brazilian fish *Thalassophryne nattereri*, there are no other predatory or pathogenic species. The archaeal Methanosarcinaceae, that possess aerolysin-like genes, are anaerobic methanogens.

### Aerolysin in the context of other domains

**Figure 4:**
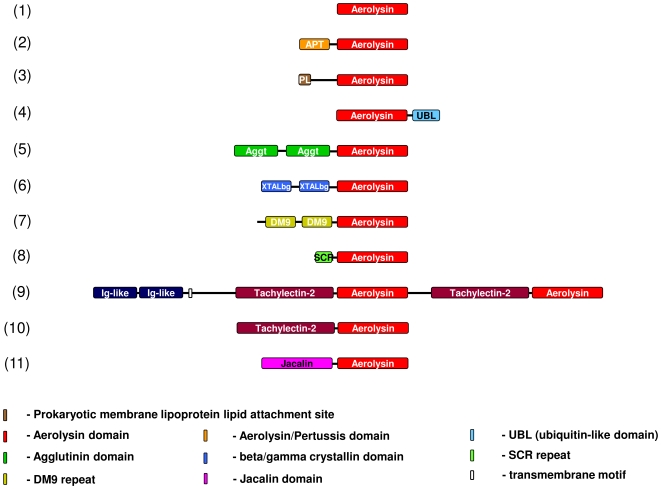
Domain organization of the Aerolysin/ETX pore-forming superfamily. Proteins 1–4 are present in bacteria, 5–11 in Eukaryotes. Domain organization representatives: (1) alpha-toxin, gi:452163 [*Clostridium septicum*]; (2) Hemolysin-3, gi:2501300 [*Aeromonas hydrophila*]; (3) hypothetical protein BACUNI_04630, gi:160892167 [*Bacteroides uniformis* ATCC 8492]; (4) pXO2-60 protein, gi: 10956450 [*Bacillus anthracis*]; (5) hypothetical protein CAN71829, gi:147838248 [*Vitis vinifera*]; (6) ep37-L2, gi:2339973 [*Cynops pyrrhogaster*]; (7) Natterin-3 precursor, gi:75571591 [*Thalassophryne nattereri*]; (8) hypothetical protein NEMVEDRAFT_v1g221281, gi:156349328 [*Nematostella vectensis*]; (9) hypothetical protein LOC494812, gi:148223884 [*Xenopus laevis*]; (10) hypothetical protein LOC613112, gi:73853870 [*Xenopus tropicalis*]; (11) hypothetical protein LOC568775, gi:162139040 [*Danio rerio*].

In our analysis we identified several domain topologies, both in Prokaryotes and Eukaryotes (Fig. 4). In bacteria the majority of aerolysins are either single pore-forming lobe or a pore-forming domain fused to an N-terminal C-lectin type structure, as found in aerolysin.

Aerolysin turns out to be amongst the most complex members. The aerolysin domain is extended N-terminally into a so-called domain 2, involved in binding to the glycan core of Glycosylphosphatidyl inositol anchored proteins [13], [14] (Fig. 2D). Fused to this GPI-binding domain is an N-terminal lectin domain [15] involved in binding to N-linked sugars present on the polypeptide moiety of GPI-anchored proteins [16].

We also identified two novel bacterial fusions. The first is the *Bacteroides uniformis* ATCC 8492 protein (gi: 160892167) from a recently sequenced *B. uniformis* genome obtained from the human gut (data obtained from the Human Microbiome Project). The unique feature of this protein is the presence of a prokaryotic membrane lipoprotein lipid attachment site at the N terminus. In Prokaryotes, membrane lipoproteins are synthesized with a precursor signal peptide, which is cleaved by a specific lipoprotein signal peptidase (signal peptidase II). The peptidase recognizes a conserved sequence and cuts upstream of a cysteine residue to which a glyceride-fatty acid lipid is attached [17]. It seems then that *Bacteroides* protein is specifically bound to the plasma membrane, which is proven by the PSORT cell location program that predicts this aerolysin-like protein to be extracellular. The other case is the *Bacillus anthracis* protein pXO2-60 (gi: 10956450; alternative names: BXB0074/GBAA_pXO2_0074) located on the pXO2 plasmid, known to be involved in virulence [18], [19]. The aerolysin domain of this protein is most similar to the *Clostridium perfringens* epsilon-toxin. While it harbors no N-terminal fusion, it carries a β-grasp fold at the C-terminus. Importantly, the *B. anthracis* gene has been shown to be highly regulated by the general virulence regulator, AtxA [20] - it is up-regulated an order of magnitude more than the anthrax toxin and capsule genes. Together with the finding that the pXO2 plasmid is indispensable for anthrax virulence [21], these data suggest that pXO2-60 protein may play an important role in *B. anthracis* cytotoxicity.

Eukaryotic proteins containing aerolysin domains can also be found in fungi. They are present in several species of pathogenic *Basidiomycota* and three *Ascomycota*. The common theme for all of them is arboreal/plant pathogenesis (*Laetiporus sulphureus*, *Agrocybe chaxingu*, *Pleurotus eryngii* var. Ferule, *Ganoderma lucidum*, *Flammulina velutipes*, *Hericium erinaceum*). The only exceptions from the rule are members of the *Coprinus* genus (*C. cinerea* and *C. comatus*) that are saprophytes. The presence of a toxin in this genus suggests that these fungi may in fact be pathogenic; another hypothesis is that the aerolysin domain is used for defense against other pathogens or when hunting. For instance, *C. comatus* is known to attack nematodes [22], [23]. Our analysis suggests it is indeed a pore-forming protein belonging to the aerolysin family. Other aerolysins from *Basidiomycota* were identified in a ‘fungal pathogens of plants’ sequencing project (Fu,M., Wu,Z., Lin,Q. and Xie,L., unpublished).

The eukaryotic aerolysin family members that seem to be most similar in structure (and function) to canonical bacterial toxins are composed of an N-terminal lectin domain followed by the pore-forming domain. We detected three new combinations of this type. In the grapevine *Vitis vinifera* and the wheat *Triticum aestivum* two N-terminal agglutinin and a C-terminal aerolysin-like domains constitute a protein that in the latter organism was shown to be involved in defense against insects and maybe other pathogens [24], [25]. Agglutinin from *Amaranthus caudatus* (amaranthin) is a lectin from the ancient South American crop, amaranth grain. Although its biological function is unknown, it can agglutinate A, B and O red blood cells, and has a carbohydrate-binding site that is specific for the methyl-glycoside of the T-antigen found linked to serine or threonine residues of cell surface glycoproteins [26]. The structure of this lectin is a beta-trefoil fold and forms a homodimer with each monomer composed of two domains [27], [28]. The authors suggest that HFR-2, the aerolysin-like protein from wheat, may normally function in defense against certain insects or pathogens. They also propose that as virulent insects (larvae) manipulate the physiology of the susceptible host; the HFR-2 protein may be forced to insert in plant cell membranes at the larvae feeding sites and by forming pores provides water, ions and other small nutritive molecules to the developing larvae [25]. We found another lectin-like fusion in two fish: *Danio rerio* and *Salmo salar* (e.g., gis: 162139040 and 209732252, respectively). Most probably the same topology would be found in other fish species when sequenced. Amazingly, *D. rerio* possesses as many as 16 copies of the gene encoding the same aerolysin-like protein. The last lectin-aerolysin topology is quite unusual and may be a virtual translation of a false CDS (gi: 148223884). We discovered it in the African clawed frog *Xenopus laevis*, but it is not present in its close relative, *Xenopus tropicalis*. This protein contains a tandem of immunoglobulin-like domains at the N terminus, followed by a transmembrane motif and a duplication of large fragments located at the C terminus. The latter is composed of a tachylectin-2-like domain and an aerolysin-like domain (gi: 165970884). In *X. tropicalis*, immunoglobulin domains are in a different ORF than the lectin-toxin tandem (gi: 171847007 and 73853870, respectively). The Japanese horseshoe crab *Tachypleus tridentatus* tachylectin-2 binds specifically to *N*-acetylglucosamine and *N*-acetylgalactosamine and is a part of the innate immunity host defense system of this crab [29] Tachylectin-2 is a protein displaying a five-bladed β-propeller structure. Tachylectin-2 exhibits five virtually identical binding sites, one in each β-sheet. The high number of binding sites within a single polypeptide chain strongly suggests the recognition of carbohydrate surface structures, possibly on microbial pathogens with a fairly high ligand density [30], however, this notion has not been proven yet. Similarly, immunoglobulin domains that are present at the N terminus are also involved in innate immunity [31]. Using TMHMM2.0 program, we show that the N-terminal portion of the protein is located inside the cell. Activity of immunoglobulin-like domains in cytoplasm have already been shown in other systems [32].

In the eukaryotic family of aerolysins we identified two exceptional fusions. First, it is a crystallin domain tandem followed by an aerolysin domain. Such topology is found in the Japanese newt *Cynops pyrrhogaster* epidermis-specific protein EP37. This organism is armed with several homologues that are present in skin, gastric epithelium and fundic glands of an adult newt and in the swimming larva [33]–[36], however the authors did not identify the C-terminal toxin domain. N-terminal domains are non-lens β/γ-crystallin domains that have a Greek key structure. Crystallins were so named when they were recognized as the proteins that provide the crystallin lens of the vertebrate eye with its indispensable transparency and unique refractive properties [37]. Because lens cells live as long as their host, crystallins also have to live long. Crystallin ancestors can be tracked to bacteria and can be used for diverse purposes, e.g. as enzymes, toxins or antistress proteins [38], [39]. The topology found in EP37 is in accordance with the canonical crystallin proteins with a tandem of crystallin motifs. In solution β-crystallins are known to form dimers whereas γ-crystallins are monomeric. β-crystallins assembly into higher order complexes: tetramers and the likely assembly of this protein in the lens is that of higher heteroligomers [40]. This conformation fits the oligomeric nature of aerolysin.

The second exceptional topology can be found in Brazilian venomous fish *Thalassophryne nattereri* proteins named natterins [e.g. gi: 75571591]. There are 5 known paralogues of this toxin with 4 full-length proteins (natterins 1–4). These are known to cause nociception and oedema [41]. Fractions containing natterin were positively tested for the kininogenase activity, but no similarity to aerolysin toxins has been proposed or shown. The N-terminal region of natterins is occupied by two DM9 domains first discovered in *Drosophila* [42], but with no function ascribed. We have discovered the similarity of twin DM9 to a half of the MFP2 protein from pig roundworm *Ascaris suum*. MFP2 increases the rate of movement *in vitro* in *Ascaris* sperm cells and appears to function by increasing the rate of the major sperm protein (MSP) polymerization, possibly in a manner analogous to formins in actin-based systems [43], [44]. Authors suggest MFP2 could increase the rate of MSP polymerization by increasing the nucleation rate, or by increasing the amount of polymerization competent MSP, or by decreasing the termination rate. MSP provides sperm locomotion by the assembly and disassembly of filaments and replaces actin in the nematode filament structure [45], [46]. Function of DM9 in natterins is unknown. We discovered a similar protein from the red jungle fowl (*Gallus gallus*)(gi: 118105776), with no function assigned to date. Interestingly, gene coding for the chicken protein overlaps head-to-tail with a chromatin modifying protein 2A (CHMP2A)(gi; 124249308). This DNA region is not assigned to any *G. gallus* chromosomes.

### Architecture of the aerolysin superfamily

**Figure 5:**
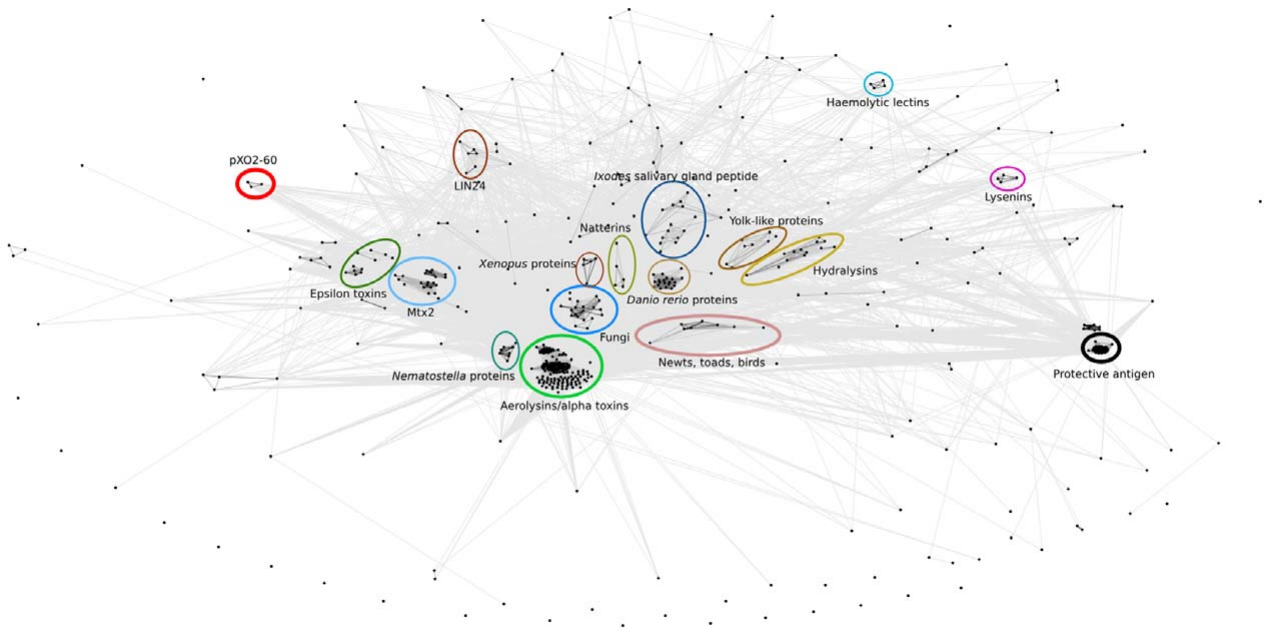
Cluster map of aerolysin superfamily. All sequenced from nr database similar to aerolysin core profile at E-value of 100 and better were used including false positives. Major groups of proteins are highlighted with ovals and described. Presence of a clear false positive (protective antigen) is highlighted to indicate difficulties in assessing the border of the superfamily.

We made a cluster map of sequences of the aerolysin superfamily (see Fig. 5) that included all hits to our profile within E-value of 100 as assessed with HMMER3 (http://hmmer.janelia.org/) (see Methods). Major groups of aerolysin-like sequences form distinct clusters and the largest cluster is understandably composed of aerolysins and alpha toxins. Other proteins are spread across several small groups, which nevertheless form a distinct cluster within the core. Several clusters are formed by toxins with known structure and recognized similarity to aerolysins, such as ETX or Mtx2 toxins, hydralysins and parasporins or insect yolk-like proteins (Fig. 5). Other groups are mainly composed of proteins of similar phylogenetic origin, which is not surprising given that they are identified based on similarity (see Methods). Distinct clusters are formed by proteins from *Nematostella*, *Cynops*, *Ixodes* or *Danio rerio*. Also plant and fungi proteins tend to form separate clusters. All these clusters form an easily recognizable supergroup with many interconnections (Fig. 5).

Interesting small groups are formed at the boundaries of the core. These distant clusters are formed by hemolytic lectins, known to have structure similar to aerolysins and ETX toxins; lysenins, pXO2-60 and anthrax protective antigen. The last one has a clearly different structure from aerolysins despite sharing a very similar mode of pore-formation [47] and as such does not belong to the aerolysin superfamily. Both the distance and number of connections were not sufficient to distinguish between a false-positive case, such as protective antigen and a true positive such as hemolytic lectin. Therefore we decided to fall back on E-value scores and assume that pXO2-60 is a border-defining member of the aerolysin superfamily.

### Structural model of pXO2-60 (aerub)

pXO2-60 is composed of three elements: signal peptide, aerolysin-domain and a ß-grasp fold, also found in ubiquitin. The signal peptide is predicted to be cleaved at position 29 (AAA-BBB). We have modeled the 3D structure of the exported part using two template structures: 1uyj (*Clostridium perfringens* epsilon toxin) and 1ubi (ubiquitin protein).

**Figure 6:**
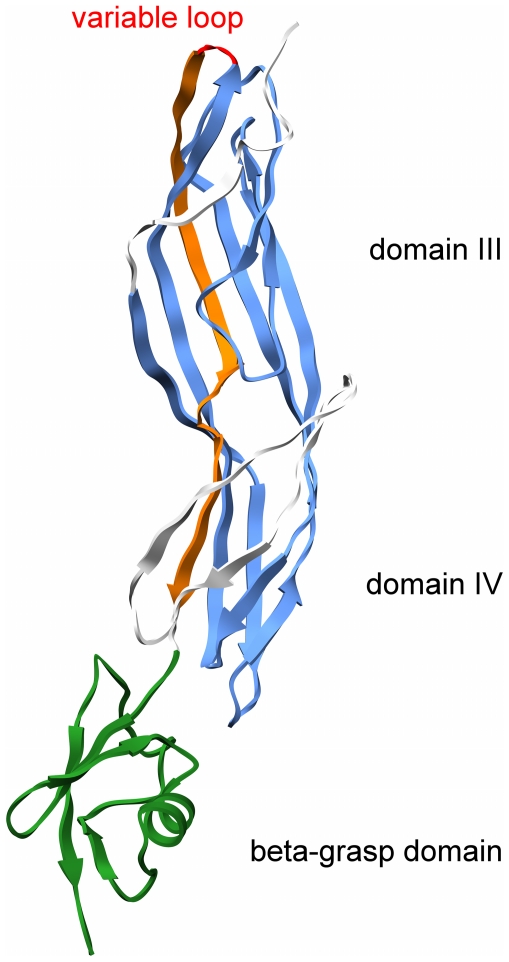
Structural model of pXO2-60. Color scheme: blue - core with conserved sequence; red – variable loop; orange – the fifth, weakly conserved β-strand; green – β-grasp domain modeled on ubiquitin protein. Two parts of the conserved core that form b-sandwich structures are marked as domain III and IV (historically). Variable loop that connects β4 and weakly conserved β5 is barely visible because of its short length.

The aerolysin domain of pXO2-60 contains the complete core of the superfamily with small additions in front of β1 and after β5 (Fig. 6). These fragments adopt the β-strand conformation and they extend two β-sandwich structures originally named domain III and domain IV in first analyses of aerolysins structures. The small β-strand is positioned between the hairpin of β4: β5 and the insertion loop. Two small β-strands that follow β5 extend β-sheets of β4-β5-β1 and β3–β4, respectively. The variable loop present in other members of the superfamily is only a few residues long in pXO2-60.

**Figure 7:**
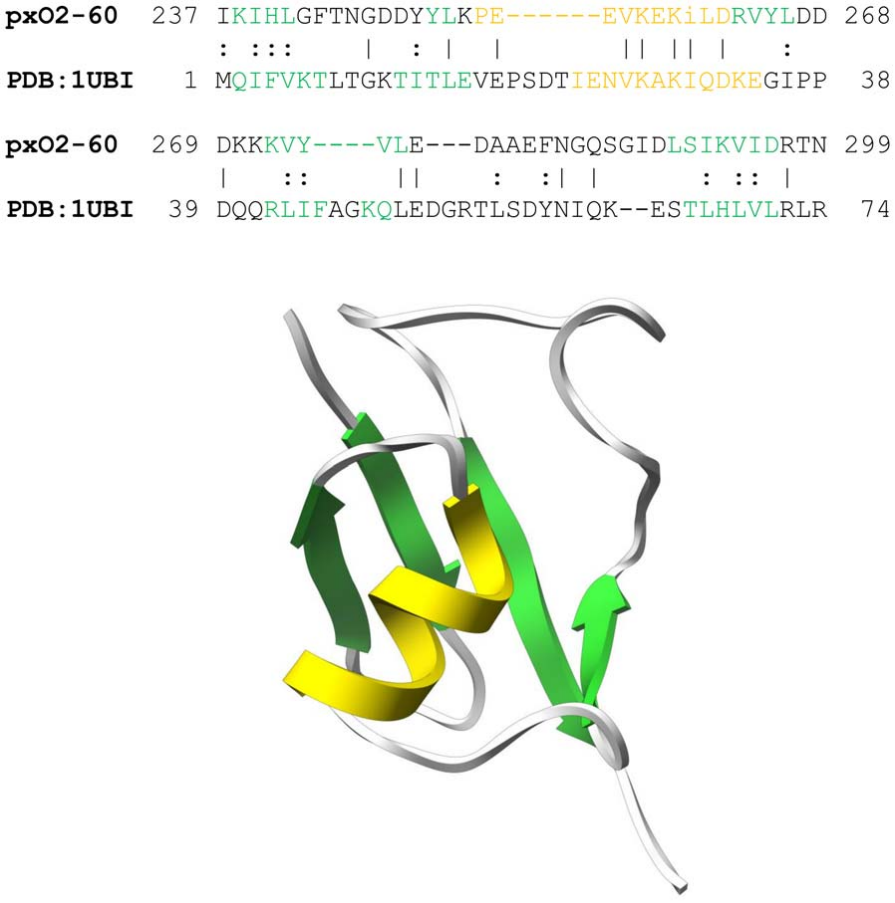
Alignment between ubiquitin fold domain of pXO2-60 and ubiquitin protein 1ubi. Green segments correspond to beta conformation, while orange to alpha conformation. Model with the same color scheme is shown below alignment.

Following aerolysin domain there is a domain that most likely adopts a β-grasp fold. We call it an ubiquitin fold domain because ubiquitin is the most prominent protein with such a scaffold. The domain was modeled on a template of ubiquitin protein (1ubi, see Fig. 7), however β-grasp fold is found in proteins of many different functions, such as translation initiation factors, immunoglobulin-binding proteins, glutamine synthetases, ferredoxin-like proteins or GTP binding proteins. It is also found at the C-terminus of staphylococcal/streptococcal toxins, where at least in some cases it mediates dimerization [48]. High structural similarity of proteins of diverse functions and sequences complicates the assignment of the C-terminal domain of pXO2-60 to any of these groups. Sequence identity at 20% and similarity at 40% over ca. 80 residues between this fragment and the closest template are not enough to clearly classify this domain within specific proteins with the β-grasp fold.

Model of pXO2-60 exemplifies the diversity of members of the aerolysin superfamily. Its compact structure, with no insertions in the variable loop, represents a new type on the structural map of the aerolysin-like fold. Presence of the ubiquitin-like domain at the C-terminus is also unique – pXO2-60 is the only protein from the superfamily with such a fusion.

## Discussion

In this study we have shown that aerolysin-like pore forming toxins share not only structural features, but also their similarity is recognizable at the sequence level. Additionally, we were able to define the real common theme for the family and have shown that it is much smaller and compact than previously thought. That information helped us to significantly increase the coverage of the family of aerolysin-domain containing proteins and find a large number of previously unknown members.

Analysis of the common structural core has shown that the functionally important unit may be as small as the amphipatic loop and the five β-strands. The fifth C-terminal β-strand that connects two halves of the complex seems to be structurally required, however its sequence does not seem to be conserved. This may be due to a variable loop immediately preceding this segment that imposes constantly changing sequence onto this segment. Other explanation could be that this β-strand must have only general properties of an extended structure because structural constrains of the β-sheet will likely keep it in place.

It is important to note that an attempt to define a common core for aerolysin-like proteins together with identification of several new members of the superfamily has been published shortly before submitting this manuscript in a review concerning *Laetiporus sulphureus* lectin and the aerolysin family [49]. While some results of that study significantly overlap with ours, its authors use traditional approach to describe the structural organization of aerolysin core (i.e. two domains).

Our analysis suggests that both prokaryotic and eukaryotic family members can be used either for attack or for defense. It is obvious in such species as bacterial pathogens *Clostridium* and *Aeromonas* or the eukaryotic Brazilian fish *Thalassophryne nattereri*. Cnidarians, newts, most probably non-pathogenic bacteria and other species may use it to defend against attackers. An uncertain point is the presence of aerolysins in some previously thought-to-be saprophytic or mycorrhizal fungi (*Coprinopsis cinerea*, *Laccaria bicolor*): are these toxins used for defense or are important in other functions (e.g. mycorrhiza)? Most puzzling case is of the honeybee anarchy 1 aerolysin-like protein, ‘a genetic locus for worker sterility in a social insect’ (cited after annotation in gi: 67848428). Its function seems to be unrelated to either attack or defense. Does it play a role in a physiological process? Is it an element in sterility regulation?

Our analysis also allowed the identification of previously unknown domain topologies. In Eukaryotes unexpected fusions include crystallin and DM9 domains. We hypothesize their functions to be similar to lectin domains, which is the facilitation of cell membrane binding. In bacteria, novel fusions were identified for proteins expressed in *Bacillus anthracis* and *Bacteroides uniformis*. The latter has an N-terminal membrane lipoprotein lipid attachment site that suggests a unique mechanism of cell attachment. *B. anthracis* pXO2-60 protein is the only one to possess a C-terminal ubiquitin fold domain. It will be of interest to determine whether this ubiquitin fold has a role on the outside of the target cell, or whether it is at some point translocated, possibly through the channel formed by the aerolysin-domain in the cytoplasm of the host cell. The determination of a precise structure, or function, of this domain would help to qualify it to anything in between a classical ubiquitin, a mechanical cap to control the aerolysin pore, an immunoglobulin-binding (Ig-binding) domain or a ligand binding domain [50]–[52]. Also it would be interesting to verify if the N-terminal segment of pXO2-60 positioned between hairpin b4–b5 and the insertion loop has similar inactivation function to parasporins where the N-terminus forms a ß-sheet with the insertion loop blocking its rearrangement and/or movement.

An interesting far-fetching hypothesis may be driven from experiments by Welkos et al. and Chand et al. that show that the lack of protective antigen does not significantly diminish the *B.anthracis* virulence in the mouse model [53]–[55]. The presence of protective capsule genes on the pXO2 plasmid may be insufficient to kill, therefore the presence of another toxin on this plasmid suggests its involvement in virulence. We hope future experiments will soon test our supposition.

## Methods

To identify aerolysin-like sequences, we made queries at the National Center for Bioinformatics Information (NCBI) using BLAST and PSI-BLAST [56], [57] on the non-redundant database (nr). Because the sensitivity of these tools is low, we have also used methods based on Hidden Markov Models. In the first step we compared the profile built on the alignment of the conserved aerolysin domain, available at the PFAM database, to the nr database with HMMER (citation) implemented as FastHMMER tool at MPI Toolkit website [58]. Based on results from this step, we have built a manual profile containing all aerolysin-like sequences and used it again against the nr database, however this time using the newly released HMMER3 package (http://hmmer.janelia.org/). We have employed an E-value of 10 in order to identify as many hits as possible and, then, we have manually analyzed the distribution to assess which threshold to apply to the results. This was necessary, as the software was still in alpha development stage. The twilight-zone hits were analyzed with HHpred [59] to obtain an external confirmation of the presence of the aerolysin domain. As a result, we have applied an E-value of 0.001 as a final threshold to hits from HMMER3. Additionally, all low-complexity sequences were removed from the final list; sequences of these hits were extracted using the Entrez service (www.ncbi.nlm.nih.gov/Entrez/). A sequence alignment was generated on the basis of pairwise alignments of the sequences and PFAM aerolysin domain using HMM-HMM comparison tool HHpred [59]. These were later manually adjusted to satisfy hydrophobic pattern of beta-strands.

For cluster map we have extracted all sequences from NCBI non-redundant database at an E-value of 100 using the hmmsearch program from HMMER3 package; that set included false positives. All vs. all comparisons were done with the jackhammer application from the same package with default of 5 iterations.

Domain annotation for selected proteins was obtained using combined effort of Interpro [60], SMART [61], Pfam [62], HHpred [59], Bioinfo.pl Metaserver [63] and FFAS03 [64] tools followed by a manual verification. No single E-value threshold was used in all cases. Presence of signal peptide was predicted using SignalP 3.0 server [65]. We have also searched for transmembrane helices with TMHMM2.0 server [66].

Both full Aerub sequence as well as single N- and C-terminal domain regions were analyzed with 3D-Jury server [63] to identify optimal templates and to derive reliable sequence-structure mappings using consensus alignment approach and 3D assessment [67]. The three dimensional model of Aerub was built with the MODELLER program [68] using as templates the structure of Clostridium perfringens epsilon-toxin (PDB code 1uyj) [8] for N-terminal Aerolisin/ETX pore-forming domain and the structure of human ubiquitin (PDB code 1ubi) [69] for C-terminal beta-grasp fold domain.

Figures of structures were prepared using Chimera [70].

## Supporting Information

Table S1 **List of proteins identified as members of the aerolysin superfamily containing Uniprot ID, protein name and species.**
(XLS)
